# Effect of transdermal estrogen dose regimen for endometrial preparation of frozen‐thawed embryo transfer on reproductive and obstetric outcomes

**DOI:** 10.1002/rmb2.12370

**Published:** 2021-02-13

**Authors:** Tatsuyuki Ogawa, Tsuyoshi Kasai, Maki Ogi, Jiro Fukushima, Shuji Hirata

**Affiliations:** ^1^ Department of Obstetrics and Gynecology Faculty of Medicine University of Yamanashi Chuo Japan; ^2^ Konohana Clinic Kai Japan

**Keywords:** constant dose, endometrial preparation, frozen‐thawed embryo transfer, hormone replacement cycle, transdermal estrogen patches

## Abstract

**Purpose:**

Previous studies have reported different methods of estrogen administration during endometrial preparation for frozen‐thawed embryo transfer (FET). This study aimed to investigate a beneficial regimen of transdermal estrogen administration for FET.

**Methods:**

We investigated the reproductive and obstetric outcomes of FET by comparing the increasing dose (ID) group that mimics changes in serum estradiol during the menstrual cycle and the constant dose (CD) group. Transdermal patches were used for estrogen administration in both groups. In our hospital, we targeted 315 cycles of the ID group in which FET was performed in 2017 and 324 cycles of the CD group in which FET was performed in 2018. In all cases, single embryo transfer was performed.

**Results:**

All were singleton pregnancies. There was no difference in clinical pregnancy rate (28.9% vs 28.2%, *P* =.837) and live birth rate (17.3% vs 21.4%, *P* =.201) between the ID and CD groups. Spontaneous abortion rate was significantly lower in the CD group than in the ID group (37.2% vs 23.0%, *P* =.041). There was no difference in obstetrical outcomes.

**Conclusions:**

It was considered that the simple CD regimen may be more beneficial than the complicated ID regimen.

## 1 INTRODUCTION

Frozen‐thawed embryo transfer (FET) is an important procedure in assisted reproductive technology (ART). According to a report by the International Committee for Monitoring Assisted Reproductive Technologies,^1^ FET pregnancies accounted for 27.0% of ART pregnancies born worldwide in 2012. This varies widely from country to country. In Japan, FET pregnancy accounts for 86.7% of 56 979 ART pregnant children born in 2018.^2^ The FET cycle regimen is roughly divided into two, the ovulatory cycle and hormone replacement cycle (HRC). In the 2017 Cochrane Review,^3^ FET cycle regimens were considered; this review did not find sufficient evidence to support the use of one cycle regimen in preference to another in preparation for FET in subfertile women with regular ovulatory cycles. However, in cases such as polycystic ovarian syndrome and premature ovarian insufficiency (POI) with ovulation disorders, HRC is useful for endometrial preparation for FET. In addition, women who are busy with work often prefer HRC to NC in order to schedule visits in advance.

The method of estrogen administration in HRC varies from report to report. First, there are differences in the administration routes such as oral, transdermal, and vaginal. It has been reported that the live birth rate (LBR) did not differ depending on the administration route.^4^, ^5^, ^6^ Next, there are regimens that mimic changes in serum estradiol during the menstrual cycle to change the dose, and fixed‐dose regimens. There are many reports suggesting conventional mimicking of the menstrual cycle,^7^, ^8^ but there are also reports suggesting that it is not necessary to vary the dose.^9^ There are few reports comparing clinical outcomes due to this difference in dosing schedules. It was reported that there was no difference in LBR by comparing the “increasing dose (ID)” group and the “constant dose (CD)” group using the oral agent and the patch.^10^

To our knowledge, there are no reports comparing obstetric outcomes depending on whether the dose of the estrogen patch is changed. At our hospital, considering thrombotic and hepatic side effects, we used a patch for estrogen administration. Until 2017, the patches were administered in ID mimicking the menstrual cycle before the start of progestin administration. However, since 2018, the patches were administered in CD. In the administration regimen ^8^ that imitates E_2_ of the menstrual cycle, the change in dose is complicated. Although the ID regimen in this study was relatively simple, but prior to this study, the number of patches pasted was sometimes incorrect. The CD regimen was extremely easy, and the patch count was never wrong. This was considered beneficial in that it reduced the stress on the patient due to infertility treatment. The CD regimen may also be useful to medical providers since it aids in the planning of HRC schedules. However, changes in the method of administration may affect reproductive and obstetric outcomes. In this study, we investigated which dose regimen of estrogen patches was more beneficial for endometrial preparation for FET.

## 2 MATERIALS AND METHODS

### 2.1 Study population and design

In our hospital, we targeted 315 cycles of the ID group, in which estrogen patches were administered by the ID regimen and HRC‐FET was performed in 2017, and 324 cycles of the CD group, in which estrogen patches were administered by the CD regimen and HRC‐FET was performed in 2018. Regarding the patients’ background, there were no between‐group differences in terms of age at the time of oocytes collection, primary infertility rate, indication of ART, primipara status, recurrent pregnancy loss (RPL) rate, body mass index (BMI), serum anti‐Müllerrian hormone (AMH) level, and number of previous ET cycles (Table 1). In all cases, suppression with a gonadotropin‐releasing hormone agonist (GnRHa) was not performed. Patients with follicular development on the 14th day of estrogen administration were excluded from this study. If an endometrial polyp measuring 1 cm or more was observed or in the case of a septate uterus, transcervical resection was performed before ART was introduced. Primary outcomes were clinical pregnancy rate (CPR) and LBR. Secondary outcomes were obstetric outcomes (preterm delivery, neonatal sex and body weight, placental weight, bleeding volume of labor, and obstetric complications). Obstetric complications included placenta accreta, placental abruption, placenta previa, preterm premature rupture of the membrane, hypertensive disorder of pregnancy (HDP), and gestational diabetes mellitus.

**TABLE 1:** Demographics of patients undergoing estrogen replacement therapy

	Total	Increasing dose	Constant dose	*P* value
n	639	315	324	
Female age (years)	36.8 ± 4.7	37.0 ± 4.8	36.6 ± 4.7	.419^a^
Primary infertility	228 (35.7)	118 (37.5)	110 (34.0)	.354^b^
Secondary infertility	411 (64.3)	197 (62.5)	214 (66.0)	
Indication of ART				
Tubal factor	58 (9.1)	29 (9.2)	29 (9.0)	.910 ^a^
Endometriosis	58 (9.1)	27 (8.6)	31 (9.6)	.661^a^
Ovulation disorder	37 (5.8)	18 (5.7)	19 (5.9)	.935^a^
Male factor	167 (26.1)	90 (28.6)	77 (23.8)	.166^a^
Unexplained infertility	260 (40.7)	120 (38.1)	140 (43.2)	.188^a^
Primipara	374 (58.5)	196 (62.2)	178 (54.9)	.061^b^
Multipara	265 (41.5)	119 (37.8)	146 (45.1)	
Recurrent pregnancy loss	88 (13.8)	44 (14.0)	44 (13.6)	.886^b^
Body mass index (kg/m^2^)	21.2 ± 2.8	21.2 ± 2.9	21.2 ± 2.8	.624^a^
Serum AMH (ng/mL)	3.2 ± 8.4	3.5 ± 12.0	2.8 ± 4.8	.201^a^
No. of previous ET cycles	2.3 ± 13.6	2.4 ± 12.2	2.3 ± 15.0	.442^a^

Note

Data are expressed as mean ± standard deviation or number (%).

Abbreviations: AMH, anti‐Müllerrian hormone; ART, assisted reproductive technology; ET, embryo transfer.

^a^ Mann‐Whitney *U* test.

^b^ Pearson's chi‐square test.

### 2.2 Hormone replacement cycle: Frozen‐thawed embryo transfer

**FIGURE 1:**
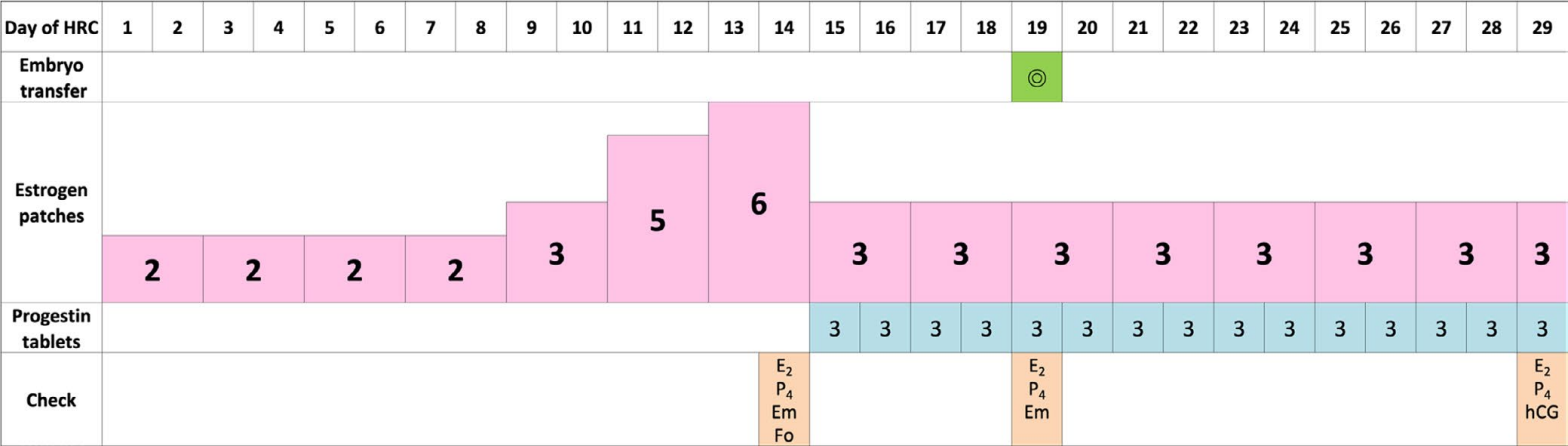
The increasing dose (ID) regimen of hormone replacement cycle for frozen‐thawed embryo transfer (FET). Estrogen patches were replaced every 2 d and were increased gradually. The thickness of the endometrium was examined on the 14th day. From the next day, progestin tablets were administered vaginally one tablet at a time every 8 h. From the start of administration of progestin, the number of estrogen patches was fixed at 3. FET was performed on the 5th day of progestin administration. HRC, hormone replacement cycle; E_2_, serum estradiol; P_4_, serum progesterone; Em, thickness of endometrium; Fo, follicle development; hCG, serum human chorionic gonadotropin

**FIGURE 2:**
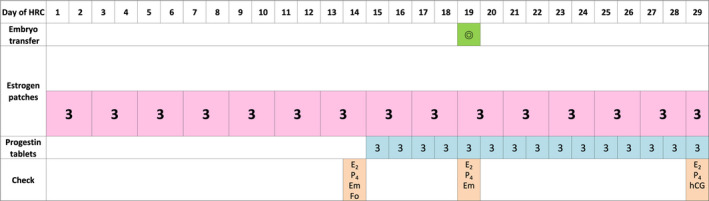
The constant dose (CD) regimen of hormone replacement cycle for frozen‐thawed embryo transfer (FET). Estrogen patches were replaced every 2 d, and the number of patches was fixed at 3 from the 1st day. The thickness of the endometrium was examined on the 14th day. From the next day, progestin tablets were administered vaginally one tablet at a time every 8 h. FET was performed on the 5th day of progestin administration. HRC, hormone replacement cycle; E_2_, serum estradiol; P_4_, serum progesterone; Em, thickness of endometrium; Fo, follicle development; hCG, serum human chorionic gonadotropin

The ID and CD regimens are shown in Figures 1 and 2, respectively. Administration of estrogen (estrogen replacement therapy; ERT) using patches (ESTRANA Tapes^®^ 0.72 mg, Hisamitsu Pharmaceutical Co., Inc) was started from the 2nd or 3rd day of the menstrual period or withdrawal bleeding due to an estrogen and progestin drug. Patches were replaced every 2 days. In the ID group, two patches on the 1st to 8th day of ERT, three patches on the 9th and 10th days, five patches on the 11th and 12th days, and six patches on the 13th and 14th days were used (22 patches in total by the 14th day). The thickness of the endometrium was examined on the 14th day. In the CD group, three patches were fixed from the 1st day of ERT, and the thickness of the endometrium was examined on the 14th day (21 patches in total by the 14th day). If the thickness of the endometrium was 7 mm or more, additional progestin tablets (LUTINUS Vaginal Tablets^®^ 100 mg; Ferring Pharmaceuticals Co., Ltd.) were administered vaginally one tablet at a time every 8 hours, starting the following day. However, if the thickness of the endometrium on the 14th day was <7 mm, the same dose of estrogen patches was continued, and the endometrium was remeasured 2 days later. In both groups, the number of estrogen patches was fixed at 3 from the start of administration of progestin.

Frozen‐thawed embryo transfer was performed on the 5th day of progestin administration. The embryos were fertilized by conventional in vitro fertilization or intracytoplasmic sperm injection and cryopreserved at Gardner classification 3BB or higher on the 5th or 6th day.^11^ All oocytes were derived from themselves but did not include doner eggs. In all cases, single embryo transfer was performed. Serum human chorionic gonadotropin (hCG) level was measured on the 15th day of progestin administration. When an increase in serum hCG level was observed (25 mIU/mL or more), a diagnosis of chemical pregnancy was made, and the day was defined as the 4th week and 0 days of pregnancy. From then, the estrogen patches and the progestin tablets were fixed at three patches and three tablets per day, respectively, which were continued until the 9th week and 6th day of the pregnancy.

## 3 RESULTS

There was no between‐group difference in terms of the follicular development rate on the 14th day of estrogen administration (Table 2). Because follicular development was observed in 14 patients in the ID group and 15 patients in the CD group, embryo transfer was canceled in 29 patients and reproductive and obstetric outcomes were examined in 301 and 309 patients, respectively. In both groups, there were no patients whose embryo transfer was canceled due to thinning of the endometrium. In the ID group, serum estradiol (E_2_) was high (*P* <.001) on the 14th day of ERT because double estrogen patches were applied to the CD group. On the day of FET, there was no difference in E_2_ because the count of patches was the same (*P* =.488). On the 14th day of ERT and the day of FET, there was no difference in the thickness of the endometrium (*P* =.089 and .881, respectively).

**TABLE 2:** Clinical outcomes of frozen‐thawed embryo transfer in hormone replacement cycle

	Total	Increasing dose	Constant dose	*P* value
n	639	315	324	
Follicular development	29 (4.5)	14 (4.4)	15 (4.6)	.910^b^
n	610	301	309	
Serum estradiol (pg/mL)				
On the 14th day of ERT	372 ± 222	465 ± 241	283 ± 157	<.001^a^
On the day of FET	285 ± 174	289 ± 172	283 ± 175	.488^a^
Endometrial thickness (mm)				
On the 14th day of ERT	10.5 ± 2.2	10.4 ± 2.2	10.6 ± 2.1	.089^a^
On the day of FET	10.3 ± 2.6	10.3 ± 2.3	10.3 ± 2.5	.881^a^
Chemical pregnancy	205 (33.6)	99 (32.9)	106 (34.3)	.711^b^
Clinical pregnancy	174 (28.5)	87 (28.9)	87 (28.2)	.837^b^
Ectopic pregnancy	1 (0.6)	1 (1.1)	0 (0)	1^c^
Spontaneous abortion	52 (30.1)	32 (37.2)	20 (23.0)	.041^b^
Artificial abortion	2 (1.2)	2 (2.3)	0 (0)	
RPOC after abortion	4 (7.7)	3 (8.8)	1 (5.0)	1^c^
Ongoing pregnancy	119 (19.5)	52 (17.3)	67 (21.7)	.169^b^
Live birth	118 (19.3)	52 (17.3)	66 (21.4)	.201^b^

Note

Data are expressed as mean ± standard deviation or number (%).

Abbreviations: ERT, estrogen replacement therapy; FET, frozen‐thawed embryo transfer; RPOC, retained products of conception.

^a^ Mann‐Whitney *U* test.

^b^ Pearson's chi‐square test.

^c^ Fisher's exact test.

All were singleton pregnancies. There was no difference in the chemical pregnancy rate and CPR (*P* =.711 and.837, respectively). Spontaneous abortion rate (SAR) was significantly lower in the CD group (*P* =.041), but there was no significant difference in LBR (*P* =.201). In the ID group, one patient had a tubular pregnancy, one patient had an artificial abortion due to a worsening mental illness, and one patient had an induced abortion because the fetus had 21 trisomy. In the CD group, one patient had an unexplained IUFD at the 26th week of pregnancy (Table 2). Obstetric outcomes were not significantly different (Table 3).

**TABLE 3:** Obstetric outcomes of ongoing pregnancy

	Total	Increasing dose	Constant dose	*P* value
Ongoing pregnancy	119	52	67	
Live birth	118	52	66	
Preterm delivery	11 (9.2)	4 (7.7)	7 (10.4)	.753^a^
Neonatal				
Male	63 (52.9)	26 (50)	37 (55.2)	.571^b^
Female	56 (47.1)	26 (50)	30 (44.8)	
Body weight (g)	3016 ± 519	3037 ± 466	3002 ± 556	.802^c^
Small for gestational age	7 (5.9)	2 (3.8)	5 (7.6)	.462^a^
Large for gestational age	18 (15.3)	7 (13.5)	11 (16.7)	.630^b^
Placental weight (g)	552 ± 124	554 ± 102	551 ± 138	.594^c^
Bleeding volume of labor				
Vaginal delivery (g)	717 ± 470	673 ± 446	746 ± 489	.479^c^
Cesarean section (g)	917 ± 378	961 ± 392	886 ± 374	.546^c^
Obstetric complications				
Placenta accreta	20 (16.9)	9 (17.3)	11 (16.4)	.897^b^
Placenta abruption	2 (1.7)	1 (1.9)	1 (1.5)	1^a^
Placenta previa	2 (1.7)	0 (0)	2 (3.0)	.503^a^
Preterm PROM	2 (1.7)	0 (0)	2 (3.0)	.503^a^
HDP	12 (10.0)	7 (13.4)	5 (7.5)	.281^b^
GDM	14 (11.7)	5 (9.6)	9 (13.4)	.521^b^

Note

Data are expressed as mean ± standard deviation or number (%).

Abbreviations: GDM, gestational diabetes mellitus; HDP, hypertensive disorder of pregnancy; PROM, premature rupture of the membrane.

^a^ Fisher's exact test.

^b^ Pearson's chi‐square test.

^c^ The Mann‐Whitney *U* test.

A subgroup analysis was performed to examine the cause of less spontaneous abortion in the CD group. Since there were more primiparas in the ID group (although there was no significant difference), the ID and CD groups were classified as primipara and multipara, respectively. Table 4 shows the sub‐analyzed reproductive outcomes (chemical pregnancy, clinical pregnancy, ongoing pregnancy, and live birth). The rate of spontaneous abortion of multiparas in the CD group was as low as 13.8%, but there was no significant difference among the 4 groups. Table 5 shows the background, E_2_ level, and endometrial thickness of patients with clinical pregnancy in the four groups. Previous ET cycles were significantly less in multiparous women than in primiparous women. Naturally, the E_2_ level on the 14th day of ERT was high in the ID group, but no other significant difference was observed.

**TABLE 4:** Sub‐analyzed reproductive outcomes of frozen‐thawed embryo transfer in hormone replacement cycle

	Increasing dose		Constant dose		*P* value
	Primipara	Multipara	Primipara	Multipara	
n	196	105	178	131	
Chemical pregnancy	66 (33.7)	33 (31.4)	66 (37.1)	39 (29.8)	.566^a^
Clinical pregnancy	58 (29.6)	29 (27.6)	57 (32.0)	30 (22.9)	.355^a^
Ectopic pregnancy	1 (1.7)	0 (0)	0 (0)	0 (0)	1^b^
Spontaneous abortion	20 (34.5)	12 (41.4)	16 (28.1)	4 (13.8)	.084^b^
Artificial abortion	0 (0)	2 (6.9)	0 (0)	0 (0)	
Ongoing pregnancy	37 (18.9)	15 (14.3)	41 (23.0)	26 (19.8)	.349^a^
Live birth	37 (18.9)	15 (14.3)	41 (23.0)	25 (19.1)	.348^a^

Abbreviations: Data are expressed as mean ± standard deviation or number (%).

^a^ Pearson's chi‐square test.

^b^ Fisher's exact test.

**TABLE 5:** Demographics and clinical outcomes of clinical pregnancy

	Increasing dose		Constant dose		*P* value
	Primipara	Multipara	Primipara	Multipara	
n	58	29	57	30	
Female age (years)	34.2 ± 5.3	36.2 ± 3.4	34.2 ± 5.3	35.0 ± 3.9	.246^a^
Recurrent pregnancy loss	4 (6.9)	4 (13.8)	2 (3.5)	1 (3.3)	.305^b^
Body mass index (kg/m^2^)	20.7 ± 2.3	20.3 ± 2.6	21.1 ± 2.9	21.4 ± 3.1	.275^a^
Recurrent pregnancy loss	4 (6.9)	4 (13.8)	2 (3.5)	1 (3.3)	.305^b^
Serum AMH (ng/mL)	4.7 ± 18.5	4.4 ± 14.2	3.9 ± 7.2	3.4 ± 3.5	.822^a^
No. of previous ET cycles	2.1 ± 5.2	0.8 ± 1.7	2.1 ± 5.5	1.3 ± 1.8	.003^a^
Serum estradiol (pg/mL)					
On the 14th day of ERT	454 ± 265	440 ± 247	279 ± 168	243 ± 124	<.001^a^
On the day of FET	278 ± 143	281 ± 128	267 ± 157	255 ± 188	.276^a^
Endometrial thickness (mm)					
On the 14th day of ERT	10.4 ± 2.2	10.8 ± 2.2	10.8 ± 2.7	10.2 ± 1.9	.745^a^
On the day of FET	10.3 ± 2.1	10.1 ± 2.3	10.7 ± 2.7	10.8 ± 2.0	.275^a^

Note

Data are expressed as mean ± standard deviation or number (%).

Abbreviations: AMH, anti‐Müllerrian hormone; ERT, estrogen replacement therapy; ET, embryo transfer; FET, frozen‐thawed embryo transfer.

^a^ Kruskal‐Wallis test.

^b^ Fisher's exact test.

## 4 DISCUSSION

The primary outcome of this study was that there was no difference in CPR and LBR between the two groups. However, there was significantly less SAR in the CD group. Since this study was retrospective, the background may have been influenced. The CD group tended to have a higher number of multiparas (*P* =.061). There were a few spontaneous abortions of multiparas in the CD group, but there was no significant difference between the four groups. In addition, the clinical pregnancy rate was low in the CD group multiparas, and as a result, the ongoing pregnancy rate and LBR were not high. The E_2_ value, endometrial thickness, and BMI of the CD group multiparas were also similar to those of the overall population. However, the number of RPLs was rather low. In a previous study, it was reported that there were no differences in CPR and LBR, but the CD group had a higher biochemical pregnancy rate than the ID group.^10^ Considering this study and previous studies, the CD regimen does not aggravate reproductive outcomes. Other confounding factors, which had not been examined in the two studies, may have an influence.

There are some reports that HRC has no difference in LBR compared with the natural cycle (NC), but can increase obstetric complications. According to a Japanese report, HRC increased significantly for gestational age, HDP, and placenta accreta compared with NC.^12^ HRC is considered a factor in high‐risk pregnancy. By examining the pathological tissue of the placenta, it has also been reported that HRC may increase hemorrhage during delivery.^13^ On the other hand, there are patients with ovulation disorder and POI, in whom endometrial preparation is difficult in NC. Therefore, research like this study that seeks a lower risk HRC is needed. The secondary outcomes of this study were obstetric outcomes, especially bleeding volume of labor and obstetric complications; however, no difference was observed between the two groups. It was considered that the change from ID to CD regimens did not have an adverse effect on obstetric outcomes.

It has been reported that transdermal administration of estrogen does not go through the liver and its effect on endometrial preparation is superior to other administration routes.^8^, ^14^, ^15^ This study used patches; although there are reports of comparisons with other routes of administration. A study comparing transdermal gels and oral preparations reported that the LBR of transdermal gels was significantly higher.^4^ However, it should be noted that the report is a small study with 15 cases of live births in the two groups. In a study comparing patches and oral preparations ^5^ and a study comparing patches and vaginal tablets,^6^ there was no difference in the ongoing pregnancy rate. In recent years, there have been few reports of using subcutaneous injection or intramuscular injection.

It has been reported that endometrial thickness can be used to evaluate whether E_2_ priming is sufficient.^16^ This study did not include cases of embryo transfer cancellation due to thinning of the endometrium. Since there was no difference in outcomes, E_2_ priming by CD was considered sufficient. It is recommended to change the dose and administration route when E_2_ priming is insufficient.^17^ It is hoped that a useful regimen will be investigated for each administration route.

When starting HRC, pretreatment with GnRHa suppression may be performed. The Cochrane review stated that the LBR was high when GnRHa was used (low‐quality evidence).^3^ However, one report suggests that there is no significant difference in reproductive outcomes with or without GnRHa.^18^ When suppression is performed, GnRHa is administered from the previous cycle of FET, so two cycles are required. Therefore, GnRHa suppression has not been performed in our hospital.

There is also a report of performing FET in an ovulatory cycle using letrozole in patients with ovulation disorder.^19^ It is considered that letrozole has a lower risk of ovarian hyperstimulation syndrome than gonadotropin does. In the ovulatory cycle using letrozole, obstetric complications may be less than HRCs like NC. However, the disadvantage of increasing the number of visits to confirm ovulation is the same as that with NC. The useful cycle regimen for FET should be selected after its strengths and weaknesses are shown.

There are several limitations to this study. First, this was a single‐center retrospective study. Hopefully, large‐scale, prospective studies will be conducted in the future. Second, preimplantation genetic testing (PGT) was not performed. PGT has become widespread in recent years. This study was conducted before the start of the clinical study of PGT in Japan.^20^ In addition, since most cases do not undergo marital chromosome testing, it is possible that cases such as balanced reciprocal translocation are included. In the future, it would be a more valuable study if it could be examined using euploid embryos by PGT. Third, the cases of uterine morphological abnormalities, fibroids, and adenomyosis were included. Lastly, antiphospholipid antibody syndrome or coagulation disorder was not considered. The above may be considered as confounding factors that affect reproductive outcomes.

In conclusion, the CD regimen was considered more beneficial than the ID regimen. In HRC‐FET using the CD regimen, LBR and obstetric outcomes were similar, whereas spontaneous abortion was lower than that observed when using the ID regimen. Further, CD has the advantage that the administration method is simple. It is hoped that further research will be conducted to consider a more beneficial regimen for endometrial preparation for FET.

## CONFLICT OF INTEREST

Tatsuyuki Ogawa, Tsuyoshi Kasai, Maki Ogi, Jiro Fukushima, and Shuji Hirata declare that they have no conflicts of interest.

## ETHICAL APPROVAL

The study was performed in accordance with the ethical standards as laid down in the 1964 Declaration of Helsinki and its later amendments or comparable ethical standards, and it was approved by the Human Ethics Review Committee of Yamanashi University Hospital (Receipt number: 1987). Informed consent for participation was obtained from all patients included in the study.
